# Editorial: Imaging of the Vestibular System

**DOI:** 10.3389/fneur.2022.937955

**Published:** 2022-06-23

**Authors:** Marianne Dieterich

**Affiliations:** Department of Neurology and German Center for Vertigo and Balance Disorders (DSGZ), University Hospital, Ludwig-Maximilians University, Munich, Germany

**Keywords:** vestibular system, labyrinth, brain network, imaging, MRI, PET, EEG, evoked potentials

The articles submitted to the Research Topic with the overarching title “*Imaging of the Vestibular System***”** can best be structured according to the following two categories:

- imaging of the peripheral system with the inner ear and eighth nerve, and
- imaging of the central circuitry from the vestibular nuclei in the pontomedullary brainstem to cerebellar midline structures and multiple cortical areas centered around the parieto-insular-opercular region.

Historically, imaging of peripheral structures within the inner ear was dominated in the 1990s by the otolaryngologist Harold Schuknecht (Pathology of the Ear. 2nd Edition, 1993, Lea and Febinger, Philadelphia) (1), who published the first experimental data on the pathophysiology of the most frequent labyrinthine disorders and proposed new creative hypotheses, which were subject to lively and sometimes controversial discussion, for example for benign peripheral positioning vertigo, vestibular neuritis, and Menière's disease. In contrast, the structure, function, and disorders of the human central vestibular system that is represented by a largely distributed, multisensory, and sensorimotor vestibular network, in particular its cortical hubs, were increasingly disclosed by imaging over the last 25 years [e.g., (2–9)]. Around a quarter of patients who present with the key symptoms of vertigo, dizziness, and imbalance are found to have central vestibular disorders.

The recent collection of articles here—with six original research articles, two systematic reviews, and one case report—focuses, on the one hand, on normal and pathological labyrinthine characteristics which may serve as indicators and reliable follow-up parameters for various forms of inner ear diseases such as endolymphatic hydrops. Precise measurement of the endolymphatic space by *in-vivo* non-invasive MRI with intravenous delayed contrast medium is increasingly becoming an essential clinical diagnostic tool to distinguish various causes of vestibulocochlear syndromes associated with endolymphatic hydrops. During the last years, different methodological approaches were used to investigate the endolymphatic space, either by intratympanal application of gadolinium or by non-invasive intravenous delayed contrast agent enhanced, high-resolution MR imaging (10–13) (Boegle et al.). The latter has the advantage of simultaneous imaging of both ears which allows a comparison of the volumetric parameters of both ears and can be helpful to differentiate Menière's disease from vestibular migraine. It turned out that an endolymphatic hydrops occurred more often in Menière's disease, and showed a higher grade and an asymmetry between both ears compared to vestibular migraine or a combined condition in which the patients fulfill the definition of both diseases (Oh et al.). To improve volumetric quantification methods further a novel open-source inner ear fluid segmentation approach was developed using a deep learning model (Ahmadi et al.). This segmentation method showed high accuracy, robustness toward domain shift, and rapid prediction times, and could speed up inner ear MRI analyses in the future. Another article deals with the relationship between imaging and electrocochleography (ECG) in 50 patients with Menière's disease (He et al.). A correlation was found between the endolymph hydrops in 3D-FLAIR MRI and ECG parameters, in particular between the area ratio of the summating potential to the action potential and the cochlear hydrops. This ECG parameter appeared sensitive and reliable for diagnosing the endolymph hydrops.

Other important structures of the inner ear are the capillaries that form a semi-permeable barrier, the blood-labyrinth barrier, less permeable than capillary barriers elsewhere. Also using MRI with intravenous contrast agents, here as a marker for the barrier's integrity, a dysfunction could probably be disclosed as a mechanism for several audio-vestibular disorders. A systematic review (Song et al.) on 14 animal studies and 53 studies in humans with different diseases found constant and reliable parameters in healthy ears of animals and humans, with a maximum contrast signal at 4 h in humans. Patients with idiopathic sudden sensorineural hearing loss and otosclerosis showed increased signal intensity before and shortly after contrast medium injection, whereas patients with Menière's disease and vestibular schwannoma showed an increased signal after 4 h. However, since sample size, control groups, and blinded analysts were heterogeneous, future studies with consistent methods are needed.

Another vascular dysfunction of the inner ear is presented in a case report of a patient with acute labyrinthine ischemia who subsequently developed a posterior semicircular canal fibrosis (Castellucci et al.).

On the other hand, three contributions deal with different aspects of the central vestibular system that is examined by MRI, PET, and vestibular-evoked potentials.

Vertigo, dizziness, double vision, and hearing loss may be symptoms of an acute ischemia or hemorrhage in the brain and/or labyrinth. About 4–10% of patients presenting with vertigo and balance disorders in the emergency room suffer from stroke and therefore need urgent diagnostics and therapy. However, symptomatology can be very similar in acute peripheral and central vestibular disorders, a reason why special neuro-otological examinations like the HINTS test (head impulse, nystagmus in different gaze positions, test for skew deviation) are performed in the acute situation for differentiation. In the acute situation, the first question is: Is it an acute unilateral peripheral or a central vestibular syndrome, and, if central, which lesion site is it? An acute peripheral vestibular syndrome can be mimicked by lesions of the medullary brainstem, cerebellar peduncle, and especially midline cerebellar structures such as the uvula, nodulus, tonsil, and the flocculus (7).

In a prospective trial examining these patients in the emergency room, symptoms and lesion topography were analyzed to characterize the lesion-symptom relationship in acute vestibular and ocular motor strokes (Zwergal et al.). An acute unilateral stroke was found in 47 of 351 patients (13%) with MRI lesions located in the cerebellar hemispheres in vertigo/dizziness, whereas strokes with double vision showed lesions in the upper brainstem. In another study, changes in the cortical networks were examined in healthy individuals who perceive self-motion after exposure to passive motion, a phenomenon named mal de debarquement syndrome (MdDS) (Jeon et al.). Here 28 fishermen, 15 of whom experienced transient symptoms of MdDS, were tested with neuropsychological assessment, vestibular testing, structural MRI, resting-state functional MRI, and FDG-PET. The detailed analyses disclosed activation of prefrontal and deactivation of cerebellar networks that correlated with neuropsychological assessment. The functional neuroimaging findings showed similarities with functional dizziness and anxiety disorders suggesting a shared mechanism of enhanced self-awareness.

In a review article on vestibular-evoked cerebral potentials (EP), results are summarized and compared from studies that have used a large range of vestibular stimulation, from natural vestibular stimulation on rotating chairs or motion platforms to artificial stimulation, for example, by sounds, galvanic stimulation or acceleration (Nakul et al.). Up to now EPs remain poorly standardized in vestibular neuroscience and neurotology.

With respect to imaging of the central vestibular system, new methods come into play such as segmentation techniques, functional connectivity, and dysconnectivity which will hopefully enable us to understand the pathophysiology of several central disorders affecting the vestibular system, e.g., vestibular migraine or cerebellar disorders. Furthermore, they can help us to understand the different types of compensatory mechanisms after an acute lesion within the network or in chronic vestibular disorders in which the other sensory and sensorimotor systems may substitute for the loss of vestibular function.

I don't want to end without noting that more recent articles on this topic of imaging the peripheral and central vestibular system can be found in specialized journals of neuroimaging and neuroscience.

## Author Contributions

The author confirms being the sole contributor of this work and has approved it for publication.

## Funding

MD was partially supported by the German Foundation of Neurology (Deutsche Stiftung Neurologie, DSN) and the German Federal Ministry of Education and Research (BMBF) via the German Center for Vertigo and Balance Disorders (DSGZ, Grant No. 01 EO 0901).

## Conflict of Interest

The author declares that the research was conducted in the absence of any commercial or financial relationships that could be construed as a potential conflict of interest.

## Publisher's Note

All claims expressed in this article are solely those of the authors and do not necessarily represent those of their affiliated organizations, or those of the publisher, the editors and the reviewers. Any product that may be evaluated in this article, or claim that may be made by its manufacturer, is not guaranteed or endorsed by the publisher.
